# 3-(2-Acetamido­eth­yl)-1*H*-indol-5-yl 4-nitro­phenyl carbonate

**DOI:** 10.1107/S1600536812038238

**Published:** 2012-09-12

**Authors:** Jan K. Maurin, Anna Zawadzka, Iwona Łozińska, Zbigniew Czarnocki

**Affiliations:** a National Medicines Institute, Chełmska 30/34, 00-725 Warsaw, Poland; b NationalCentre for Nuclear Research, 05-400 Otwock-Świerk, Poland; c Faculty of Chemistry, Warsaw University, Pasteura 1, 02-093 Warsaw, Poland

## Abstract

In the title mol­ecule, C_19_H_17_N_3_O_6_, the indole ring system is essentially planar (r.m.s. deviation = 0.009 Å) and forms a dihedral angle of 31.96 (9)° with the nitro-substituted benzene ring. In the crystal, mol­ecules are linked by pairs of N—H⋯O hydrogen bonds, forming inversion dimers which are connected by further N—H⋯O hydrogen bonds into a two-dimensional network parallel to (102).

## Related literature

For background to and potential applications of the title compound, see: Freer & McKillop (1996 ▶); Um *et al.* (2006 ▶, 2008 ▶); Gray *et al.* (1977 ▶); Zawadzka *et al.* (2012 ▶).
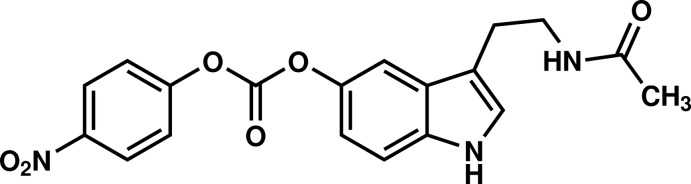


## Experimental

### 

#### Crystal data

C_19_H_17_N_3_O_6_
*M* *_r_* = 383.36
Monoclinic, 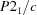

*a* = 12.3678 (3) Å
*b* = 5.0537 (1) Å
*c* = 29.1554 (6) Å
β = 92.071 (2)°
*V* = 1821.11 (7) Å^3^
*Z* = 4
Cu *K*α radiation
μ = 0.89 mm^−1^
*T* = 293 K
0.40 × 0.10 × 0.07 mm

#### Data collection

Oxford Diffraction Xcalibur Ruby diffractometer
Absorption correction: analytical (*CrysAlis PRO*; Oxford Diffraction, 2010 ▶) *T*_min_ = 0.802, *T*_max_ = 0.956
16657 measured reflections
3397 independent reflections
2413 reflections with *I* > 2σ(*I*)
*R*_int_ = 0.034

#### Refinement

*R*[*F* ^2^ > 2σ(*F* ^2^)] = 0.044
*wR*(*F* ^2^) = 0.138
*S* = 1.06
3397 reflections
260 parameters
H atoms treated by a mixture of independent and constrained refinement
Δρ_max_ = 0.22 e Å^−3^
Δρ_min_ = −0.18 e Å^−3^

### 

Data collection: *CrysAlis PRO* (Oxford Diffraction, 2010 ▶); cell refinement: *CrysAlis PRO*; data reduction: *CrysAlis PRO*; program(s) used to solve structure: *SHELXS97* (Sheldrick, 2008 ▶); program(s) used to refine structure: *SHELXL97* (Sheldrick, 2008 ▶); molecular graphics: *SHELXTL* (Sheldrick, 2008 ▶) and *PLATON* (Spek, 2009 ▶); software used to prepare material for publication: *SHELXL97*.

## Supplementary Material

Crystal structure: contains datablock(s) global, I. DOI: 10.1107/S1600536812038238/lh5522sup1.cif

Structure factors: contains datablock(s) I. DOI: 10.1107/S1600536812038238/lh5522Isup2.hkl

Supplementary material file. DOI: 10.1107/S1600536812038238/lh5522Isup3.cml

Additional supplementary materials:  crystallographic information; 3D view; checkCIF report

## Figures and Tables

**Table 1:** Hydrogen-bond geometry (Å, °)

*D*—H⋯*A*	*D*—H	H⋯*A*	*D*⋯*A*	*D*—H⋯*A*
N1—H1⋯O1^i^	0.87 (2)	2.01 (2)	2.882 (2)	177 (2)
N12—H12⋯O6^ii^	0.77 (3)	2.54 (3)	3.207 (3)	147 (3)

Symmetry codes: (i) 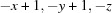; (ii) 
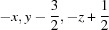
.

**Fig. 1.**
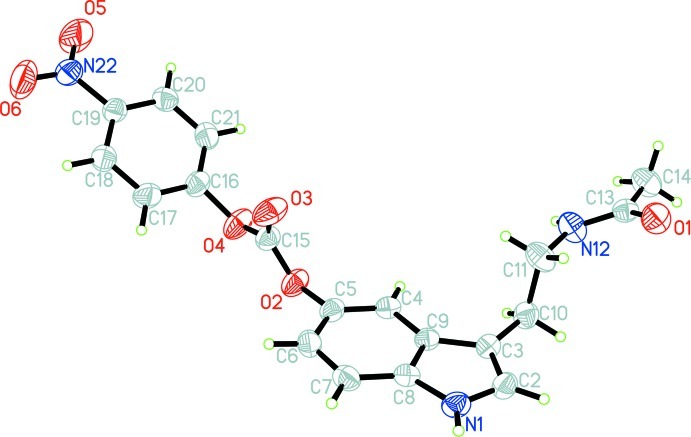
The molecular structure of the title compound with 30% dispalcement ellipsoids.

**Fig. 2.**
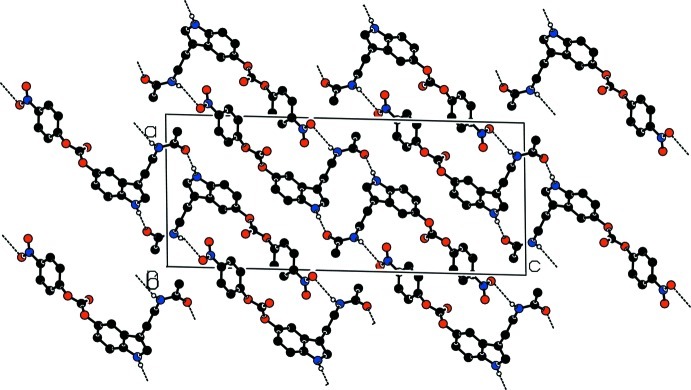
Part of the crystal structure with hydrogen bonds shown as dshed lines.

## supplementary crystallographic information

### Comment

The title compound is one of the aromatic carbonates, which constitute an important class of esters which facilitate the synthesis of a carbamate bond in the nucleofilic substitution reaction of carbonate derivatives with amines (Freer & McKillop, 1996; Um *et al.*, 2006;2008). It is also a derivative which has potential use in peptide synthesis (Gray *et al.*, 1977). We have used the title compound in the synthesis of novel tacrine-melatonine heterodimers (Zawadzka, *et al.*, 2012).

The title compound consists of three major planar fragments and a large flexible substituent having several degrees of freedom. The planar fragments are the *p*-nitrophenyl fragment, carbonate group and indole group. The r.m.s. deviations of in-plane atoms for the respective planes are 0.006, 0.009 and 0.009 Å, respectively. The carbonate group forms a dihedral angle of 80.54 (8)° with the nitro-substituted benzene ring and forms a dihedral angle of 73.23 (6)° with the indole ring system. The nitro group is slightly rotated from the plane of the attached benzene ring with a dihedral angle of 8.0 (4)°. In general the bond lengths and angles have expected values. The benzene ring may affected by anistropic displacement causing slightly shorter than expected bond lengths to be observed. In the crystal, molecules are linked by a pair of N—H···O hydrogen bonds to forms inversion dimers which are connected by further N—H···O hydrogen bonds to form a two-dimensional network parallel to (102) (Table 1 and Fig. 2).

### Experimental

To a solution of *N*-[2-(5-hydroxy-1*H*-indol-3-yl)ethyl]acetamide (0.9 g, 4 mmol) in N-methylomorfoline (0.92 ml, 8 mmol) 4-nitrophenyl chloroformate (1.61 g, 8 mmol) dissolved in 1 ml of tetrahydrofuran was added. The reaction mixture was stirred under argon for 1 h at room temperature. Evaporation of the solvents gave a residue that was purified by silica gel chromatography using a mixture of methylene chloride/methanol 95:5 as eluent to produce the title compound (0.92 g, 60%) as a yellow solid. Crystals suitable for X-ray analysis were obtained by slow evaporation of a solution of the title compound in a methylene chloride/methanol/diethyl ether mixture.

### Refinement

H atoms bonded to C atoms were placed in calculated positions with distances in the range 0.93-0.97Å and inlcuded in the refinement with U_iso_(H) = 1.2U_eq_(C) or 1.5U_eq_(C_methyl_). The positional parameters of the H atoms bonded to N atoms were refined independently with U_iso_(H) = 1.2U_eq_(N).

### Crystal data

**Table:**

C_19_H_17_N_3_O_6_	*F*(000) = 800
*M**_r_* = 383.36	*D*_x_ = 1.398 Mg m^−^^3^
Monoclinic, *P*2_1_/*c*	Melting point: 426 K
Hall symbol: -P 2ybc	Cu *K*α radiation, λ = 1.54178 Å
*a* = 12.3678 (3) Å	Cell parameters from 5868 reflections
*b* = 5.0537 (1) Å	θ = 3.0–70.0°
*c* = 29.1554 (6) Å	µ = 0.89 mm^−^^1^
β = 92.071 (2)°	*T* = 293 K
*V* = 1821.11 (7) Å^3^	Parallelepiped, colourless
*Z* = 4	0.40 × 0.10 × 0.07 mm

### Data collection

**Table:**

Oxford Diffraction Xcalibur Ruby diffractometer	3397 independent reflections
Radiation source: Enhance (Cu) X-ray Source	2413 reflections with *I* > 2σ(*I*)
Graphite monochromator	*R*_int_ = 0.034
Detector resolution: 10.4922 pixels mm^-1^	θ_max_ = 70.2°, θ_min_ = 3.0°
φ and ω scans	*h* = −15→14
Absorption correction: analytical (*CrysAlis PRO*; Oxford Diffraction, 2010)	*k* = −6→5
*T*_min_ = 0.802, *T*_max_ = 0.956	*l* = −35→35
16657 measured reflections	

### Refinement

**Table:**

Refinement on *F*^2^	Secondary atom site location: difference Fourier map
Least-squares matrix: full	Hydrogen site location: inferred from neighbouring sites
*R*[*F*^2^ > 2σ(*F*^2^)] = 0.044	H atoms treated by a mixture of independent and constrained refinement
*wR*(*F*^2^) = 0.138	*w* = 1/[σ^2^(*F*_o_^2^) + (0.0799*P*)^2^ + 0.0677*P*] where *P* = (*F*_o_^2^ + 2*F*_c_^2^)/3
*S* = 1.06	(Δ/σ)_max_ < 0.001
3397 reflections	Δρ_max_ = 0.22 e Å^−^^3^
260 parameters	Δρ_min_ = −0.18 e Å^−^^3^
0 restraints	Extinction correction: *SHELXL97* (Sheldrick, 2008), Fc^*^=kFc[1+0.001xFc^2^λ^3^/sin(2θ)]^-1/4^
Primary atom site location: structure-invariant direct methods	Extinction coefficient: 0.0014 (3)

### Special details

**Table:**

Geometry. All e.s.d.'s (except the e.s.d. in the dihedral angle between two l.s. planes) are estimated using the full covariance matrix. The cell e.s.d.'s are taken into account individually in the estimation of e.s.d.'s in distances, angles and torsion angles; correlations between e.s.d.'s in cell parameters are only used when they are defined by crystal symmetry. An approximate (isotropic) treatment of cell e.s.d.'s is used for estimating e.s.d.'s involving l.s. planes.
Refinement. Refinement of *F*^2^ against ALL reflections. The weighted *R*-factor *wR* and goodness of fit *S* are based on *F*^2^, conventional *R*-factors *R* are based on *F*, with *F* set to zero for negative *F*^2^. The threshold expression of *F*^2^ > σ(*F*^2^) is used only for calculating *R*-factors(gt) *etc*. and is not relevant to the choice of reflections for refinement. *R*-factors based on *F*^2^ are statistically about twice as large as those based on *F*, and *R*- factors based on ALL data will be even larger.

### Fractional atomic coordinates and isotropic or equivalent isotropic displacement parameters (Å^2^)

**Table:**

	*x*	*y*	*z*	*U*_iso_*/*U*_eq_	
O1	0.23003 (13)	0.0684 (3)	−0.05411 (5)	0.0836 (5)	
O2	0.33006 (13)	0.5304 (3)	0.23175 (5)	0.0741 (4)	
O3	0.22723 (14)	0.8918 (4)	0.21929 (6)	0.0915 (5)	
O4	0.20616 (14)	0.6121 (3)	0.27802 (5)	0.0842 (5)	
O5	−0.16478 (18)	1.1179 (6)	0.38600 (9)	0.1471 (10)	
O6	−0.0422 (2)	1.3762 (6)	0.40406 (10)	0.1530 (11)	
N1	0.59607 (13)	0.6072 (4)	0.08575 (6)	0.0646 (5)	
H1	0.6490 (19)	0.708 (5)	0.0772 (8)	0.078*	
C2	0.55303 (17)	0.4095 (4)	0.05894 (7)	0.0639 (5)	
H2	0.5788	0.3603	0.0306	0.077*	
C3	0.46756 (15)	0.2941 (4)	0.07904 (6)	0.0567 (5)	
C4	0.38506 (14)	0.4030 (4)	0.15764 (6)	0.0548 (4)	
H4	0.3320	0.2725	0.1570	0.066*	
C5	0.39637 (15)	0.5732 (4)	0.19355 (6)	0.0589 (5)	
C6	0.47530 (17)	0.7688 (4)	0.19661 (6)	0.0664 (5)	
H6	0.4795	0.8803	0.2220	0.080*	
C7	0.54735 (16)	0.7973 (4)	0.16196 (7)	0.0651 (5)	
H7	0.6010	0.9264	0.1635	0.078*	
C8	0.53720 (14)	0.6267 (4)	0.12471 (6)	0.0545 (4)	
C9	0.45587 (14)	0.4308 (3)	0.12168 (6)	0.0516 (4)	
C10	0.39484 (17)	0.0829 (4)	0.05951 (7)	0.0687 (5)	
H10A	0.4337	−0.0181	0.0371	0.082*	
H10B	0.3756	−0.0369	0.0839	0.082*	
C11	0.2944 (2)	0.1911 (5)	0.03725 (8)	0.0799 (6)	
H11A	0.2584	0.3023	0.0591	0.096*	
H11B	0.3137	0.3018	0.0116	0.096*	
N12	0.21938 (17)	−0.0102 (4)	0.02072 (6)	0.0786 (6)	
H12	0.193 (2)	−0.092 (6)	0.0395 (9)	0.094*	
C13	0.19251 (15)	−0.0581 (4)	−0.02296 (7)	0.0610 (5)	
C14	0.1130 (2)	−0.2769 (5)	−0.03169 (8)	0.0854 (7)	
H14A	0.0931	−0.3526	−0.0030	0.128*	
H14B	0.1451	−0.4107	−0.0502	0.128*	
H14C	0.0497	−0.2079	−0.0475	0.128*	
C15	0.25425 (16)	0.7027 (4)	0.24022 (6)	0.0622 (5)	
C16	0.13438 (17)	0.7780 (4)	0.30026 (6)	0.0651 (5)	
C19	−0.00262 (16)	1.0523 (4)	0.35211 (6)	0.0634 (5)	
C17	0.17387 (18)	0.9802 (5)	0.32675 (8)	0.0799 (6)	
H17	0.2471	1.0224	0.3268	0.096*	
C18	0.10491 (17)	1.1217 (5)	0.35341 (8)	0.0755 (6)	
H18	0.1303	1.2604	0.3718	0.091*	
C20	−0.04309 (17)	0.8522 (5)	0.32501 (7)	0.0733 (6)	
H20	−0.1164	0.8112	0.3246	0.088*	
C21	0.02650 (19)	0.7129 (5)	0.29846 (7)	0.0746 (6)	
H21	0.0010	0.5767	0.2796	0.090*	
N22	−0.07505 (16)	1.1922 (5)	0.38248 (7)	0.0800 (5)	

### Atomic displacement parameters (Å^2^)

**Table:**

	*U*^11^	*U*^22^	*U*^33^	*U*^12^	*U*^13^	*U*^23^
O1	0.0793 (10)	0.0984 (12)	0.0736 (9)	−0.0193 (9)	0.0110 (8)	0.0123 (8)
O2	0.0842 (10)	0.0792 (10)	0.0602 (8)	0.0241 (8)	0.0195 (7)	0.0118 (7)
O3	0.0920 (12)	0.0983 (12)	0.0861 (10)	0.0374 (10)	0.0293 (9)	0.0307 (9)
O4	0.1024 (12)	0.0781 (10)	0.0746 (9)	0.0228 (9)	0.0374 (8)	0.0140 (8)
O5	0.0776 (13)	0.193 (3)	0.174 (2)	−0.0145 (15)	0.0503 (14)	−0.065 (2)
O6	0.1153 (18)	0.178 (2)	0.168 (2)	−0.0113 (17)	0.0420 (15)	−0.102 (2)
N1	0.0525 (9)	0.0721 (11)	0.0699 (10)	−0.0088 (8)	0.0111 (8)	0.0046 (8)
C2	0.0630 (11)	0.0703 (12)	0.0592 (10)	0.0014 (10)	0.0122 (9)	−0.0020 (9)
C3	0.0562 (10)	0.0567 (10)	0.0576 (10)	0.0025 (8)	0.0050 (8)	−0.0009 (8)
C4	0.0488 (9)	0.0544 (10)	0.0612 (10)	0.0031 (8)	0.0038 (8)	0.0063 (8)
C5	0.0579 (11)	0.0652 (12)	0.0540 (9)	0.0131 (9)	0.0062 (8)	0.0061 (9)
C6	0.0728 (13)	0.0678 (13)	0.0580 (10)	0.0089 (10)	−0.0057 (10)	−0.0093 (9)
C7	0.0572 (11)	0.0632 (12)	0.0740 (12)	−0.0049 (9)	−0.0084 (9)	−0.0039 (10)
C8	0.0457 (9)	0.0583 (11)	0.0595 (10)	0.0013 (8)	0.0006 (8)	0.0045 (8)
C9	0.0481 (9)	0.0506 (10)	0.0561 (9)	0.0035 (7)	0.0009 (7)	0.0053 (8)
C10	0.0713 (13)	0.0622 (12)	0.0725 (12)	−0.0021 (10)	0.0037 (10)	−0.0093 (10)
C11	0.0842 (15)	0.0723 (14)	0.0817 (13)	−0.0159 (12)	−0.0164 (12)	0.0089 (12)
N12	0.0816 (13)	0.0863 (13)	0.0676 (11)	−0.0300 (10)	−0.0025 (9)	0.0143 (10)
C13	0.0520 (10)	0.0632 (12)	0.0683 (12)	−0.0015 (9)	0.0056 (9)	0.0048 (9)
C14	0.0823 (15)	0.0786 (15)	0.0941 (16)	−0.0193 (12)	−0.0118 (13)	0.0035 (13)
C15	0.0627 (12)	0.0681 (12)	0.0561 (10)	0.0062 (10)	0.0077 (9)	0.0027 (10)
C16	0.0730 (13)	0.0677 (12)	0.0556 (10)	0.0080 (10)	0.0150 (9)	0.0057 (9)
C19	0.0586 (11)	0.0780 (13)	0.0541 (10)	0.0041 (10)	0.0086 (8)	0.0017 (9)
C17	0.0549 (12)	0.0915 (16)	0.0941 (15)	−0.0066 (11)	0.0153 (11)	−0.0101 (14)
C18	0.0642 (13)	0.0838 (15)	0.0788 (13)	−0.0071 (11)	0.0077 (10)	−0.0170 (12)
C20	0.0568 (12)	0.0888 (15)	0.0744 (12)	−0.0066 (11)	0.0018 (10)	−0.0064 (12)
C21	0.0761 (14)	0.0806 (14)	0.0668 (12)	−0.0041 (12)	−0.0020 (11)	−0.0101 (11)
N22	0.0657 (12)	0.1020 (15)	0.0731 (11)	0.0047 (10)	0.0143 (9)	−0.0058 (11)

### Geometric parameters (Å, º)

**Table:**

O1—C13	1.216 (2)	C10—C11	1.485 (3)
O2—C15	1.309 (2)	C10—H10A	0.9700
O2—C5	1.423 (2)	C10—H10B	0.9700
O3—C15	1.176 (2)	C11—N12	1.447 (3)
O4—C15	1.351 (2)	C11—H11A	0.9700
O4—C16	1.397 (2)	C11—H11B	0.9700
O5—N22	1.180 (3)	N12—C13	1.327 (3)
O6—N22	1.186 (3)	N12—H12	0.77 (3)
N1—C2	1.365 (3)	C13—C14	1.496 (3)
N1—C8	1.375 (2)	C14—H14A	0.9600
N1—H1	0.87 (2)	C14—H14B	0.9600
C2—C3	1.359 (3)	C14—H14C	0.9600
C2—H2	0.9300	C16—C17	1.361 (3)
C3—C9	1.434 (3)	C16—C21	1.373 (3)
C3—C10	1.495 (3)	C19—C20	1.367 (3)
C4—C5	1.358 (3)	C19—C18	1.375 (3)
C4—C9	1.397 (3)	C19—N22	1.464 (3)
C4—H4	0.9300	C17—C18	1.375 (3)
C5—C6	1.390 (3)	C17—H17	0.9300
C6—C7	1.378 (3)	C18—H18	0.9300
C6—H6	0.9300	C20—C21	1.372 (3)
C7—C8	1.389 (3)	C20—H20	0.9300
C7—H7	0.9300	C21—H21	0.9300
C8—C9	1.412 (3)		
			
C15—O2—C5	118.89 (15)	C10—C11—H11B	108.8
C15—O4—C16	118.74 (16)	H11A—C11—H11B	107.7
C2—N1—C8	108.58 (16)	C13—N12—C11	125.66 (19)
C2—N1—H1	122.9 (15)	C13—N12—H12	119 (2)
C8—N1—H1	128.3 (15)	C11—N12—H12	115 (2)
C3—C2—N1	111.14 (17)	O1—C13—N12	122.12 (19)
C3—C2—H2	124.4	O1—C13—C14	121.85 (19)
N1—C2—H2	124.4	N12—C13—C14	116.02 (19)
C2—C3—C9	105.76 (16)	C13—C14—H14A	109.5
C2—C3—C10	127.51 (18)	C13—C14—H14B	109.5
C9—C3—C10	126.58 (17)	H14A—C14—H14B	109.5
C5—C4—C9	117.69 (17)	C13—C14—H14C	109.5
C5—C4—H4	121.2	H14A—C14—H14C	109.5
C9—C4—H4	121.2	H14B—C14—H14C	109.5
C4—C5—C6	123.53 (18)	O3—C15—O2	129.37 (19)
C4—C5—O2	117.43 (18)	O3—C15—O4	125.00 (19)
C6—C5—O2	118.76 (17)	O2—C15—O4	105.54 (17)
C7—C6—C5	119.90 (18)	C17—C16—C21	122.0 (2)
C7—C6—H6	120.0	C17—C16—O4	119.5 (2)
C5—C6—H6	120.0	C21—C16—O4	118.1 (2)
C6—C7—C8	117.76 (18)	C20—C19—C18	122.57 (19)
C6—C7—H7	121.1	C20—C19—N22	119.18 (19)
C8—C7—H7	121.1	C18—C19—N22	118.21 (19)
N1—C8—C7	130.94 (18)	C16—C17—C18	119.6 (2)
N1—C8—C9	107.17 (16)	C16—C17—H17	120.2
C7—C8—C9	121.89 (17)	C18—C17—H17	120.2
C4—C9—C8	119.21 (16)	C19—C18—C17	118.1 (2)
C4—C9—C3	133.44 (17)	C19—C18—H18	121.0
C8—C9—C3	107.35 (16)	C17—C18—H18	121.0
C11—C10—C3	112.69 (18)	C19—C20—C21	118.8 (2)
C11—C10—H10A	109.1	C19—C20—H20	120.6
C3—C10—H10A	109.1	C21—C20—H20	120.6
C11—C10—H10B	109.1	C20—C21—C16	119.0 (2)
C3—C10—H10B	109.1	C20—C21—H21	120.5
H10A—C10—H10B	107.8	C16—C21—H21	120.5
N12—C11—C10	113.74 (19)	O5—N22—O6	120.6 (2)
N12—C11—H11A	108.8	O5—N22—C19	119.8 (2)
C10—C11—H11A	108.8	O6—N22—C19	119.6 (2)
N12—C11—H11B	108.8		
			
C8—N1—C2—C3	−0.6 (2)	C9—C3—C10—C11	79.3 (3)
N1—C2—C3—C9	0.2 (2)	C3—C10—C11—N12	−176.18 (19)
N1—C2—C3—C10	175.94 (18)	C10—C11—N12—C13	−113.3 (3)
C9—C4—C5—C6	−1.1 (3)	C11—N12—C13—O1	−0.1 (4)
C9—C4—C5—O2	−174.95 (15)	C11—N12—C13—C14	−180.0 (2)
C15—O2—C5—C4	−111.9 (2)	C5—O2—C15—O3	3.7 (3)
C15—O2—C5—C6	74.0 (2)	C5—O2—C15—O4	−179.55 (17)
C4—C5—C6—C7	0.0 (3)	C16—O4—C15—O3	−14.3 (3)
O2—C5—C6—C7	173.75 (17)	C16—O4—C15—O2	168.83 (18)
C5—C6—C7—C8	0.4 (3)	C15—O4—C16—C17	−75.7 (3)
C2—N1—C8—C7	−179.0 (2)	C15—O4—C16—C21	111.2 (2)
C2—N1—C8—C9	0.7 (2)	C21—C16—C17—C18	1.4 (4)
C6—C7—C8—N1	−179.87 (19)	O4—C16—C17—C18	−171.5 (2)
C6—C7—C8—C9	0.4 (3)	C20—C19—C18—C17	−1.0 (4)
C5—C4—C9—C8	1.8 (2)	N22—C19—C18—C17	176.4 (2)
C5—C4—C9—C3	−179.15 (19)	C16—C17—C18—C19	−0.1 (4)
N1—C8—C9—C4	178.68 (16)	C18—C19—C20—C21	0.8 (3)
C7—C8—C9—C4	−1.6 (3)	N22—C19—C20—C21	−176.6 (2)
N1—C8—C9—C3	−0.6 (2)	C19—C20—C21—C16	0.5 (3)
C7—C8—C9—C3	179.20 (17)	C17—C16—C21—C20	−1.5 (3)
C2—C3—C9—C4	−178.88 (19)	O4—C16—C21—C20	171.38 (19)
C10—C3—C9—C4	5.4 (3)	C20—C19—N22—O5	6.0 (4)
C2—C3—C9—C8	0.2 (2)	C18—C19—N22—O5	−171.5 (3)
C10—C3—C9—C8	−175.54 (18)	C20—C19—N22—O6	−175.5 (3)
C2—C3—C10—C11	−95.5 (3)	C18—C19—N22—O6	7.0 (4)

### Hydrogen-bond geometry (Å, º)

**Table:**

*D*—H···*A*	*D*—H	H···*A*	*D*···*A*	*D*—H···*A*
N1—H1···O1^i^	0.87 (2)	2.01 (2)	2.882 (2)	177 (2)
N12—H12···O6^ii^	0.77 (3)	2.54 (3)	3.207 (3)	147 (3)

Symmetry codes: (i) −*x*+1, −*y*+1, −*z*; (ii) −*x*, *y*−3/2, −*z*+1/2.
